# Artificial-intelligence-assisted CCTA quantifies sex differences in coronary atherosclerotic burden at low atheroma volumes^☆^

**DOI:** 10.1016/j.ijcha.2025.101758

**Published:** 2025-07-28

**Authors:** Zoee D’Costa, Ronald P. Karlsberg, Geoffrey W. Cho

**Affiliations:** a David Geffen School of Medicine at the University of California Los Angeles, United States; b Cedars-Sinai Heart Institute, United States; c Cardiovascular Research Foundation of Southern California, United States

**Keywords:** Artificial intelligence, Coronary computed tomography angiography, Coronary atheroma volume, Sex differences, Non-calcified plaque, Plaque composition, Cardiovascular risk stratification

## Abstract

**Background:**

Coronary artery disease (CAD) manifests differently between sexes, with data suggesting females develop more non-calcified plaques that traditional calcium-centric tools may not detect.

**Methods:**

We conducted a retrospective cohort study of 100 individuals with low total atheroma volume (TAV) < 250 mm^3^ using artificial intelligence (AI)-enabled coronary computed tomography angiography (CCTA) to assess sex-based differences in coronary plaque composition. Plaque subtypes included calcified, non-calcified, and low-density non-calcified atheroma volumes.

**Results:**

Females had significantly lower total (p = 0.018) and non-calcified plaque (p < 0.001) burden compared to males. Calcified (p = 0.52) and low-density non-calcified (p = 0.16) plaque volumes did not differ significantly. Age was a consistent predictor of plaque volume across most subtypes.

**Conclusions:**

Despite low overall plaque burden, males demonstrated a higher non-calcified plaque burden than females. This finding contrasts with previous literature and underscores the potential of AI-enabled CCTA to detect subclinical coronary disease, particularly in low-risk cohorts. These results support the use of comprehensive plaque profiling in both sexes to improve early risk stratification.

## Highlights

• AI-based CCTA was used to analyze coronary plaque in a low-risk cohort (TAV < 250 mm^3^).
• Males had higher total and non-calcified plaque volumes than females.
• No significant differences were found in calcified or low-density plaque by sex.
• These findings challenge traditional assumptions about early CAD risk in females.
• AI-driven plaque characterization enhances detection of early subclinical atherosclerosis.

## Graphical abstract

**Figure:**
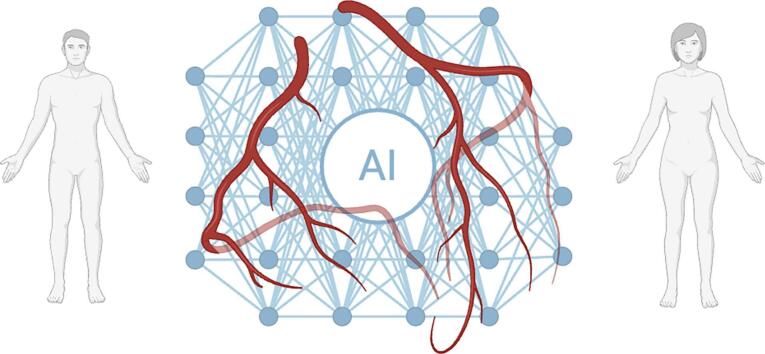
This visual summary highlights the use of artificial intelligence (AI) to analyze coronary computed tomography angiography (CCTA) in a low-risk cohort (TAV < 250 mm^3^). A male and female figure represent the study population, with AI-enhanced imaging illustrating the differential plaque composition. Males demonstrated significantly greater total and non-calcified atheroma volume than females, while calcified and low-density plaque volumes were comparable. This underscores the potential of AI-CCTA to uncover subclinical sex-based differences.

## 1 Introduction

Cardiovascular disease (CVD), particularly coronary artery disease (CAD) remains the leading cause of morbidity and mortality worldwide, with significant differences in presentation, progression and outcomes between males and females [[1], [2], [3]]. While invasive coronary angiography remains an end-stage diagnostic option and the gold standard for evaluating coronary artery disease (CAD), it does not allow for comprehensive, quantitative characterization of plaque throughout the entire coronary tree. Noninvasive imaging modalities—particularly coronary computed tomography angiography (CCTA) and coronary artery calcium (CAC) scoring—play a central role in early detection, risk stratification, treatment, and prevention. The European Society of Cardiology (ESC) guidelines recommend noninvasive imaging, such as CCTA or CAC, for patients with suspected anginal symptoms to guide diagnosis and management. Similarly, the American Heart Association (AHA) and American College of Cardiology (ACC) recommend CCTA as a Class I, Level of Evidence A diagnostic modality in patients with stable chest pain and intermediate risk of coronary artery disease, supported by high-quality evidence from multiple randomized trials. Despite these strong endorsements, these tools remain underutilized in routine risk assessment of asymptomatic patients at low to intermediate risk for CAD [[1], [2], [3], [4], [5], [6]].

Coronary artery calcium (CAC) scoring has long been a cornerstone of atherosclerotic cardiovascular disease (ASCVD) risk assessment. Calculated using non-contrast CT scans and the Agatston method, CAC scoring quantifies coronary calcification based on plaque area and peak density in Hounsfield units (HU). Risk is then categorized by CAC score: 0 (no calcification), 1–99 (mild), 100–399 (moderate), and ≥400 (severe). As an established alternative, calcified atheroma volume (CAV)—a continuous, quantitative measure derived from contrast-enhanced CCTA—captures the volumetric burden of coronary calcification. With the aid of artificial intelligence (AI) platforms (e.g. Cleerly), CAV can be computed alongside total (TAV), non-calcified, and low-density plaque volumes, enabling comprehensive, reproducible characterization of coronary atherosclerosis [5]. Importantly, CAV categories have been proposed that mirror the CAC framework, allowing for clinical analogy to CAC: 0 mm^3^ (analogous to CAC = 0), 1–99 mm^3^ (mild calcification), CAV 100–399 mm^3^ (moderate), CAV ≥ 400 mm^3^ (severe calcification) [7]. These CAV categories offer a more anatomically grounded, volumetric assessment of coronary calcification while maintaining conceptual consistency with traditional CAC thresholds.

This distinction is particularly useful when evaluating sex-based differences in CAD. Studies suggest that females tend to present with non-calcified or mixed-morphology plaques earlier in the disease course, which may still harbor high risk, placing them at risk of underdiagnosis when stratification relies solely on calcified plaque and can lead to a higher likelihood of experiencing myocardial infarction (MI) [[8], [9], [10], [11], [12]]. In contrast, males with similar risk often develop calcified lesions earlier, leading to earlier risk identification using traditional calcium-centric metrics [[13], [14], [15]]. Even among patients with low or zero TAV and CAV, high-risk non-calcified plaque features, such as low-attenuation plaque, luminal stenosis, positive remodeling, and spotty calcification, can be present and clinically relevant [5]. CCTA with AI-based quantification allows for earlier detection, and therefore, preventative and therapeutic intervention, of these features. Otherwise, they may have gone unrecognized using conventional risk assessment tools, particularly in females, a population for whom CCTA has been endorsed by expert consensus [7,11].

This retrospective cohort study aims to compare coronary plaque composition between females and males with low total atheroma volume, using AI-enabled CCTA analysis. By focusing on patients with TAV < 250 mm^3^—a group analogous to those traditionally considered “low risk” by CAC scoring—we assess differences in total, calcified, non-calcified, and low-density plaque burden to explore whether females harbor disproportionately high-risk, non-calcified disease despite minimal calcified burden. This work highlights the diagnostic limitations of calcification-centric metrics and underscores the clinical utility of CAV as a sex-sensitive, AI-derived measure of early coronary atherosclerosis. Understanding these sex-based differences in plaque composition may refine risk assessment strategies and guide personalized preventive interventions in patients classified as low risk by CAC alone [16].

## 2 Methods

**Fig. 1:**
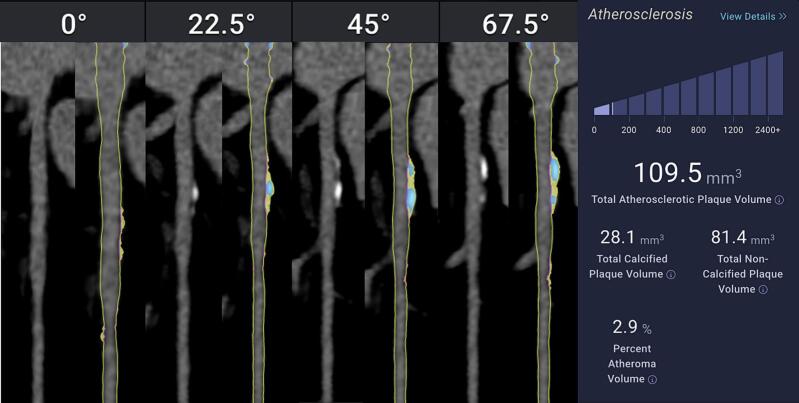
AI-Based CCTA Plaque Analysis in a Male Patient with Low Total Atheroma Volume**.** Example of plaque analysis by Cleerly, Inc CCTA software in a 50-year-old male with low total coronary atheroma volume (109.5 mm^3^). The left-sided images are of the proximal Left Anterior Descending Artery in different views of 0, 22.5, 45, and 67.5 degrees, with the plain CCTA image to the left of the AI analyzed image on the right. The images on the right are straightened MPR with color plaque overlay displaying the distribution of different plaque volumes, with yellow displaying Non-Calcified Atheroma Volume (NCAV 81.4  mm^3)^, and blue displaying Calcified Atheroma Volume (CAV 28.1  mm^3^). The patient's total percent atheroma volume (TAV) was 2.9 %. Compared with the female in Fig. 2, there is more NCAV than CAV in this male patient with similar age and TAV, a paradoxical finding compared to what generally appears in the literature.

**Fig. 2:**
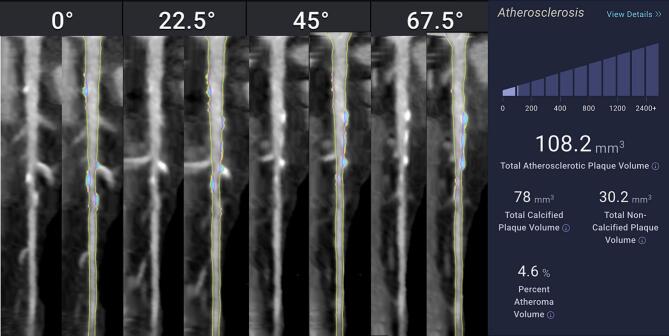
AI-Based CCTA Plaque Analysis in a Female Patient with Low Total Atheroma Volume. Example of plaque analysis by Cleerly, Inc CCTA software in a 53-year-old female with low total coronary atheroma volume (108.2 mm^3^). The left-sided images are of the proximal Left Anterior Descending Artery in different views of 0, 22.5, 45, and 67.5 degrees, with the plain CCTA image to the left of the AI analyzed image on the right. The images on the right are straightened MPR with color plaque overlay displaying the distribution of different plaque volumes, with yellow displaying Non-Calcified Atheroma Volume (NCAV 30.2  mm^3)^, and blue displaying Calcified Atheroma Volume (CAV 78  mm^3^). The patient's total percent atheroma volume (TAV) was 4.6 %. Compared with the male in Fig. 1, there is more CAV than NCAV in this female patient with similar age and TAV, a paradoxical finding compared to what generally appears in the literature.

We conducted a cross-sectional analysis of 100 random individuals with <250 mm^3^ to evaluate sex-based differences in plaque characteristics out of 1468 total patients evaluated with Cleerly software. All patients underwent clinically indicated coronary CT angiography with iodinated contrast and ECG-gating using Cleerly’s proprietary and FDA-clearned machine learning algorithm which generates an AI-created 3D model of patient’s coronary arteries and quantifies plaque. Cleerly ISCHEMIA’s algorithm uses measurements relying on invasive FFR data to determine the likelihood of vessel-level ischemia. The tool applies convolutional neural networks to segment the coronary vasculature, classify plaque types (calcified, non-calcified, low-density), and compute volumetric plaque burden in mm3. All AI outputs were reviewed for technical quality, and only studies with fully evaluable coronary segments were included in the final analysis and applied to all patient’s scans. Patients’ demographics were matched for similar risk factors. Volumetric plaque measures included total atheroma volume (TAV), calcified atheroma volume (CAV), non-calcified atheroma volume (NCAV), and low-density non-calcified atheroma volume (LD-NCAV), quantified in mm^3^ (Fig. 1 and Fig. 2). No identified additional high-risk plaque features including positive remodeling, spotty calcifications or Hounsfield Units < 30 were noted by the software in these patients, though the software is able to make these identifications. To ensure analysis of biologically relevant disease, unadjusted comparisons were limited to individuals with non-zero values across all plaque categories.

Sex differences were assessed using two-tailed Welch’s t-tests comparing females to males. To account for confounding by age, we constructed multivariable linear regression models with each plaque measure as the dependent variable and age and sex as independent variables. Sex was encoded as a binary variable (0 = female, 1 = male). Model performance was evaluated using R2.

## 3 Results

In unadjusted analyses restricted to individuals with non-zero plaque burden, females exhibited significantly lower total atheroma volume compared to males (mean difference, p = 0.018), as well as substantially lower non-calcified plaque volume (p < 0.001). No statistically significant differences were observed in calcified plaque volume (p = 0.52) or low-density non-calcified plaque volume (p = 0.16) between sexes.

**Fig. 3:**
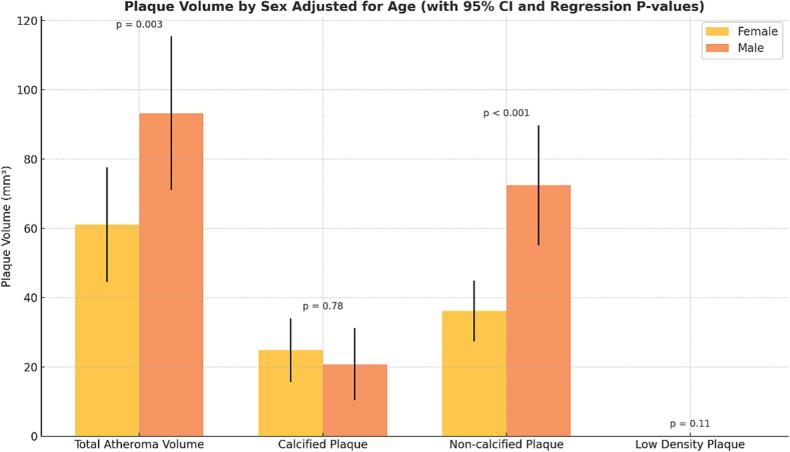
Sex-Based Differences in Coronary Plaque Volume by Subtype with Age Adjustment. Mean coronary plaque volumes (mm^3^) by sex across four plaque subtypes—total atheroma volume (TAV), non-calcified plaque (NCAV), calcified plaque (CAV), and low-density non-calcified plaque (LD-NCAV)—with error bars representing 95 % confidence intervals. Plaque volumes are adjusted for age using multivariable linear regression. Statistical significance is denoted by p-values derived from the regression model**s:** TAV (p = 0.003), NCAV (p < 0.001), CAV (p = 0.78), and LD-NCAV (p = 0.11)**.** Males exhibited significantly greater TAV and NCAV compared to females, while no sex-based differences were observed for CAV or LD-NCAV. These age-adjusted findings underscore distinct sex-related patterns in early coronary plaque development, particularly in non-calcified components.

Multivariable regression analyses, adjusting for age, confirmed these findings. Male sex was independently associated with significantly greater total atheroma volume (β = 37.4 mm^3^, p = 0.003) and non-calcified plaque volume (β = 39.3 mm^3^, p < 0.001), suggesting a disproportionate burden of overall and potentially vulnerable plaque in males. In contrast, sex was not significantly associated with calcified plaque volume (β = –1.82 mm^3^, p = 0.78) or low-density non-calcified plaque volume (β = 0.06 mm^3^, p = 0.11), highlighting that more stable or advanced plaque components may be distributed similarly across sexes (Fig. 3).

Age was a consistent predictor of plaque burden across most subtypes. In adjusted models, each additional year of age was associated with a 2.02 mm^3^ increase in total plaque volume (p < 0.001), 1.18 mm^3^ increase in calcified plaque (p < 0.001), and 0.84 mm^3^ increase in non-calcified plaque volume (p = 0.012). Age was not significantly associated with low-density non-calcified plaque (β = –0.0005 mm^3^, p = 0.71). Model explanatory power was modest for TAV, CAV, and NCAV (R^2^ ≈ 0.20) but minimal for LD-NCAV (R^2^ = 0.03).

These findings suggest that while plaque accumulation increases with age regardless of sex, males may experience a disproportionate burden of non-calcified and total plaque components, more closely associated with vulnerable or early-stage atherosclerosis.

## 4 Study limitations

Limitations include the caveats of any retrospective study, as well as the focus on artificial intelligence plaque analysis compared to human readers, though the integrity of A.I. driven CCTA plaque quantification has been well validated [17]. Other studies have shown varying sex results depending on patient presentation (e.g anginal symptoms versus those that are asymptomatic), and thus larger-scale studies are needed to clarify these differences [12,17]. In addition, the lack of sex difference in calcified or low-density plaque implies that these features may reflect more advanced or stabilized disease processes that converge across sexes with aging. While Plaque volume is important, it is not exhaustive as a measure of atherosclerotic risk. Functional data, lesion location, and compositional features such as fibrous cap integrity or inflammatory markers are not assessed by our analysis and can be expanded upon in further iterations of this study. While Cleerly’s software has the ability to analyze coronary territory data and can display both qualitative and quantitative calcification of the coronary arterial tree, this study did not analyze territory-specific data as there was not enough data for the sample size selected and will be pursued in future analyses. Finally, our smaller sample size can be expanded on in further studies to better understand the power of this phenomenon. Collectively, these results highlight the important need for further exploration of sex-specific mechanisms in atherosclerotic plaque development and vulnerability.

## 5 Discussion

This retrospective cohort study aimed to understand sex-based differences in coronary plaque burden among patients with low total atheroma volume (TAV < 250 mm^3^) using AI-enabled CCTA software (Cleerly, Inc). Based on prior literature highlighting the higher prevalence of non-calcified, high-risk plaque in females, we hypothesized that females would exhibit greater high-risk or non-calcified and low-density plaque volumes compared to males, despite minimal total plaque burden in both groups [[8], [9], [10], [11]]. However, our findings differed from our hypothesis. We found that males had significantly higher total and non-calcified plaque volumes, even given low total atheroma volume, while low-density plaque volumes and calcified atheroma volumes did not differ significantly by sex.

The analysis of calcified atheroma volume (CAV) revealed no significant difference between males and females, consistent with prior work showing that early atherosclerosis in females may not yet manifest as calcified disease [[9], [10]]. This supports the known limitation of calcium-centric risk tools, such as coronary artery calcium (CAC) scoring, in female populations [[7], [8],^10^]. Yet, contrary to our expectations, non-calcified plaque burden was higher in males, raising important questions about how atherosclerosis evolves across sexes in early-stage or subclinical disease.

This contrast with existing literature may be attributable to several key factors. First, our study focused on a cohort with low total plaque burden, in contrast to several prior studies that examined slightly different populations of symptomatic individuals or those with intermediate-to-high risk profiles [10,16]. It is possible that among asymptomatic or earlier-stage patients, males may accumulate more total and non-calcified plaque before calcification becomes prominent, while females develop vulnerable plaque more diffusely or at a comparatively later stage/timeline. Second, our analysis used AI-based quantitative CCTA rather than qualitative or manual assessments [5,7,14]. The AI platform may offer a more sensitive or reproducible delineation of plaque morphology, especially for non-calcified features that have traditionally been underestimated. Third, age and hormonal status may have played a role. Although age was adjusted for in our models, subtle age-related shifts in plaque phenotype may not have been fully captured, and we lacked detailed data on menopausal status or hormone use, both of which can influence plaque composition [8,13]. Given these complexities, a larger prospective trial with matched controls and a more diverse, adequately powered sample will be critical to clarify these sex-specific patterns in plaque phenotype and progression, especially among asymptomatic or early-stage patients. Such studies would enable deeper understanding of underlying mechanisms and help refine sex-sensitive diagnostic and preventive strategies.

While the regression models demonstrated significant associations between male sex and both total and non-calcified atheroma volumes, the moderate explanatory power (R^2^ ∼ 0.20) suggests that other unmeasured variables likely contribute to plaque burden such as lifestyle, metabolic, inflammatory, and genetic factors. These components may have influenced the distribution and phenotype of plaque in our cohort. Further prospective studies with matched controls and larger volumes are indicated to further clarify these findings.

## 6 Conclusions

Among patients with total atheroma volume <250 mm^3^, males exhibited greater total and non-calcified plaque burden than females—a finding that diverged from our initial hypothesis and from several previous studies. These results suggest that sex differences in atherosclerosis may vary depending on the stage of disease, symptom status, and the imaging modalities used. While calcified plaque burden did not differ, AI-enabled CCTA revealed a disproportionate burden of non-calcified plaque in males, even in the context of low total disease burden.

This contrast between our original expectations and observed findings highlights the importance of context-specific risk assessment and the evolving role of AI-based CCTA in refining plaque characterization. Future research should evaluate whether these early non-calcified differences translate into differential cardiovascular outcomes and explore how sex, age, and imaging modality interact to shape our understanding of coronary disease risk. Importantly, clinicians and researchers should recognize the limitations of calcification-based metrics and consider comprehensive plaque profiling, particularly in populations with low traditional risk but measurable atheroma burden.

## 7 Glossary

### 7.1 Atheroma volume (AV)

Quantitative measure of plaque burden within the coronary arteries. Includes both calcified and non-calcified components and is typically reported in cubic millimeters (mm^3^).

### 7.2 Total atheroma volume (TAV)

Total volume of all atherosclerotic plaque (calcified, non-calcified, and low-density) present in the coronary arteries.

### 7.3 Calcified atheroma volume (CAV)

Volume of plaque that has undergone calcification, typically considered more stable and representing later-stage atherosclerosis.

### 7.4 Non-calcified atheroma volume (NCAV)

Plaque that has not yet calcified. Often considered more vulnerable or unstable, and more common in early-stage atherosclerosis or among females.

### 7.5 Low-density non-calcified atheroma volume (LD-NCAV)

Subset of non-calcified plaque with particularly low Hounsfield unit density (<30 HU), often associated with high-risk, lipid-rich, or necrotic core plaques.

### 7.6 Coronary artery calcium (CAC) score

Measure of calcified plaque in the coronary arteries obtained via non-contrast CT scan, calculated using the Agatston method. Commonly categorized as: 0: No calcification, 1–99: Mild, 100–399: Moderate, ≥400: Severe.

### 7.7 Coronary computed tomography angiography (CCTA)

Non-invasive imaging modality that visualizes coronary arteries using contrast-enhanced CT scanning. It allows assessment of both calcified and non-calcified plaque, vessel remodeling, and luminal narrowing.

### 7.8 Cleerly software

Artificial intelligence-based platform used to analyze CCTA images and provide automated quantification of plaque types and volumes.

### 7.9 Artificial intelligence (AI)

Set of computational methods that enable machines to perform tasks that typically require human intelligence. In this study, AI was used to detect and quantify coronary plaque characteristics.

### 7.10 Hounsfield units (HU)

Scale used in CT imaging to measure radiodensity. In plaque characterization, HU values help differentiate between calcified and non-calcified components.

### 7.11 Sex differences in CAD

Refers to variations in presentation, plaque morphology, and clinical outcomes of coronary artery disease between males and females. Many of the references used women and females interchangeably. Our paper used female to discuss sex-based difference for specificity.

### 7.12 Subclinical atherosclerosis

Atherosclerosis that is present without causing overt symptoms or clinical events. Often detected using imaging tools such as CCTA or CAC scoring.

### 7.13 Plaque morphology

Structural and compositional features of atherosclerotic plaque, including aspects such as calcification, lipid content, fibrous cap, and vulnerability to rupture.

## Disclosures and acknowledgement of grant support

None.

## Statement of procedures and consent statement

All procedures were performed in compliance with relevant laws and institutional guidelines and have been approved by the appropriate institutional committees, (IRB Protocol 00051198, SSU0014810, 4/2022). The privacy rights of human subjects have been observed and informed consent was obtained for experimentation with human subjects. Ethics committee approval and informed consent have been obtained for studies involving patients.

## Declaration of generative AI and AI-assisted technologies in the writing process

Statement: During the preparation of this work the author(s) used ChatGPT in order to improve the readability, language, and gramma of the manuscript. After using this tool/service, the author(s) reviewed and edited the content as needed and take(s) full responsibility for the content of the published article.

## CRediT authorship contribution statement

**Zoee D’Costa:** Writing – review & editing, Writing – original draft, Visualization, Validation, Resources, Methodology, Investigation, Formal analysis, Data curation, Conceptualization. **Ronald P. Karlsberg:** Writing – review & editing, Visualization, Validation, Data curation, Conceptualization. **Geoffrey W. Cho:** Writing – review & editing, Validation, Supervision, Software, Resources, Methodology, Investigation, Formal analysis, Conceptualization.

## Funding

None declared.

## Declaration of competing interest

The authors declare that they have no known competing financial interests or personal relationships that could have appeared to influence the work reported in this paper.
